# Supplementary figures and images for: A rapid and easy-to-use spinal muscular atrophy screening tool based on primers with high specificity and amplification efficiency for SMN1 combined with single-stranded tag hybridization assay
Source: PLoS One. 2024 Aug 1;19(8):e0308179. doi: 10.1371/journal.pone.0308179 (PMC11293695; doi:10.1371/journal.pone.0308179)

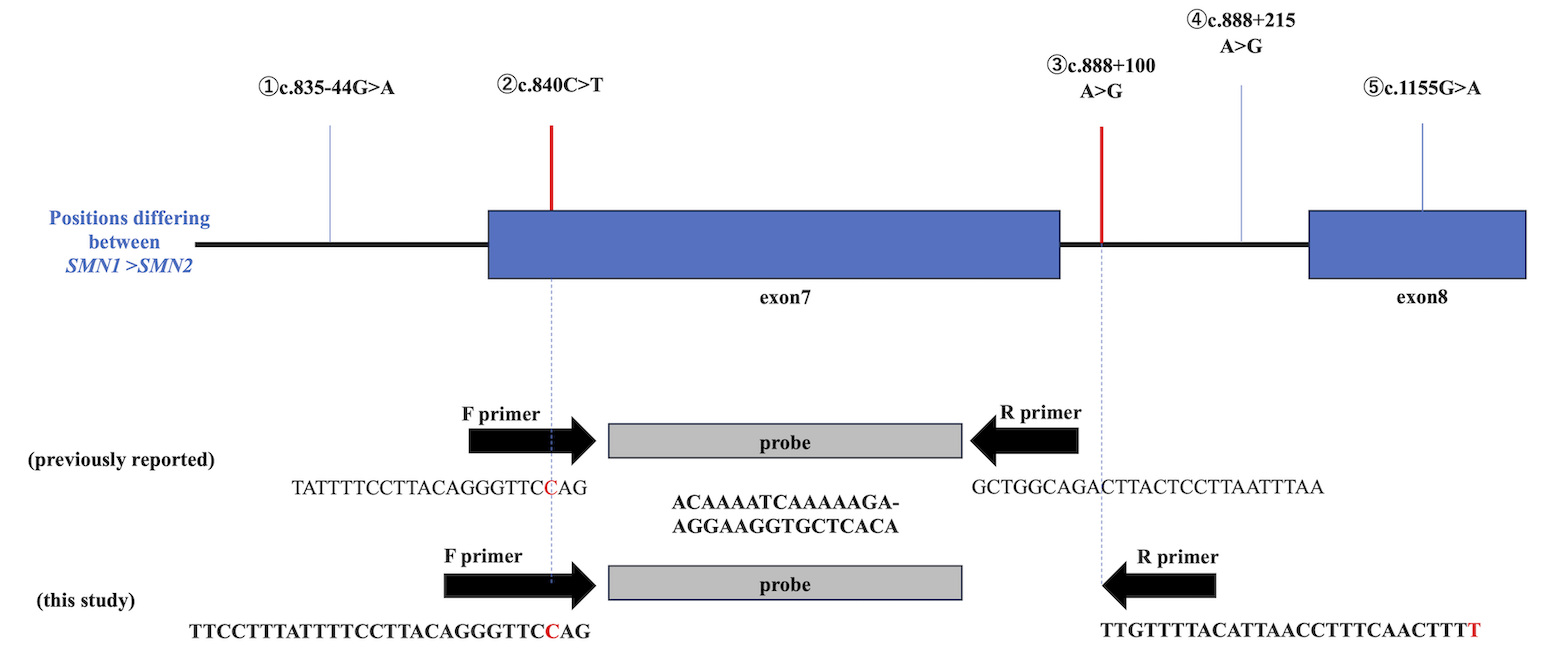

Supplement: S1 Fig — The five different nucleotides between the SMN1 and SMN2 exon7 peri-regions are shown (SMN1>SMN2). Both forward primers shown here target c.840 cite, while the reverse primer designed in this study also targets c.888+100 cite. The red-letter bases of the primers are complementary to the different bases between the two genes. (TIFF) [file pone.0308179.s001.tiff]

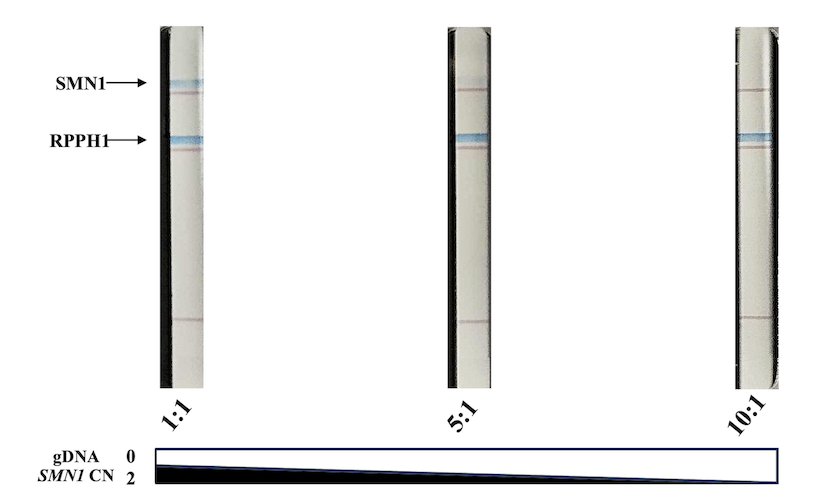

Supplement: S2 Fig — From left to right, the mixing ratios of gDNA(SMN1 CN 0) and gDNA(SMN1 CN2) are 1:1, 5:1 and 10:1. (TIFF) [file pone.0308179.s002.tiff]
