# Supplementary material for: A rapid and easy-to-use spinal muscular atrophy screening tool based on primers with high specificity and amplification efficiency for SMN1 combined with single-stranded tag hybridization assay
Source: PLoS One. 2024 Aug 1;19(8):e0308179. doi: 10.1371/journal.pone.0308179 (PMC11293695; doi:10.1371/journal.pone.0308179)
Supplement: S1 Raw images — All raw gel images in the main and supplemental figures of the manuscript are shown, with the loading order, the identity of the experimental samples, and the method used to capture the images. (PDF) [file pone.0308179.s004.pdf]

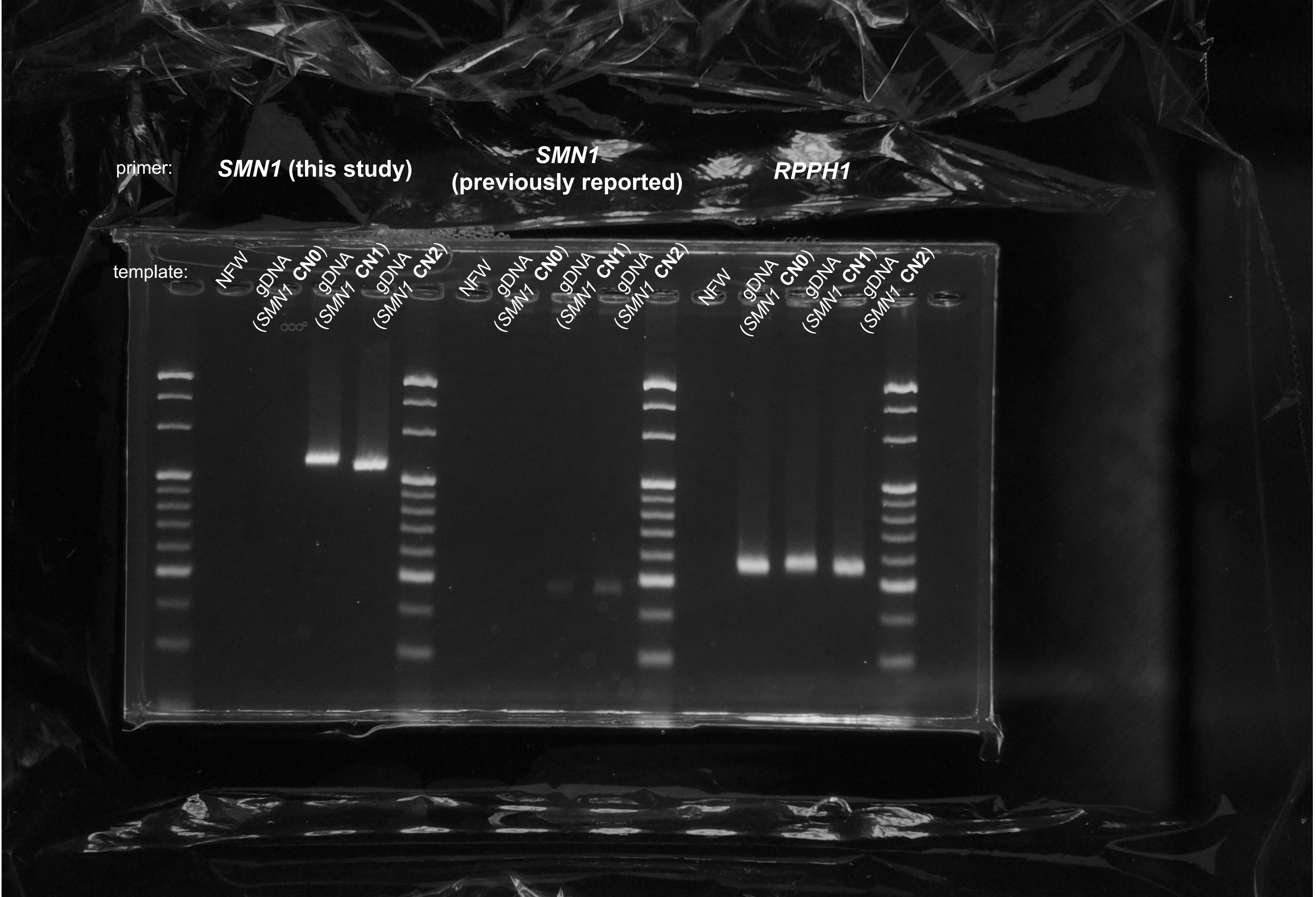

The original gel image of Figure 2(a), captured using a FAS-BG LED BOX (with exposure 10).

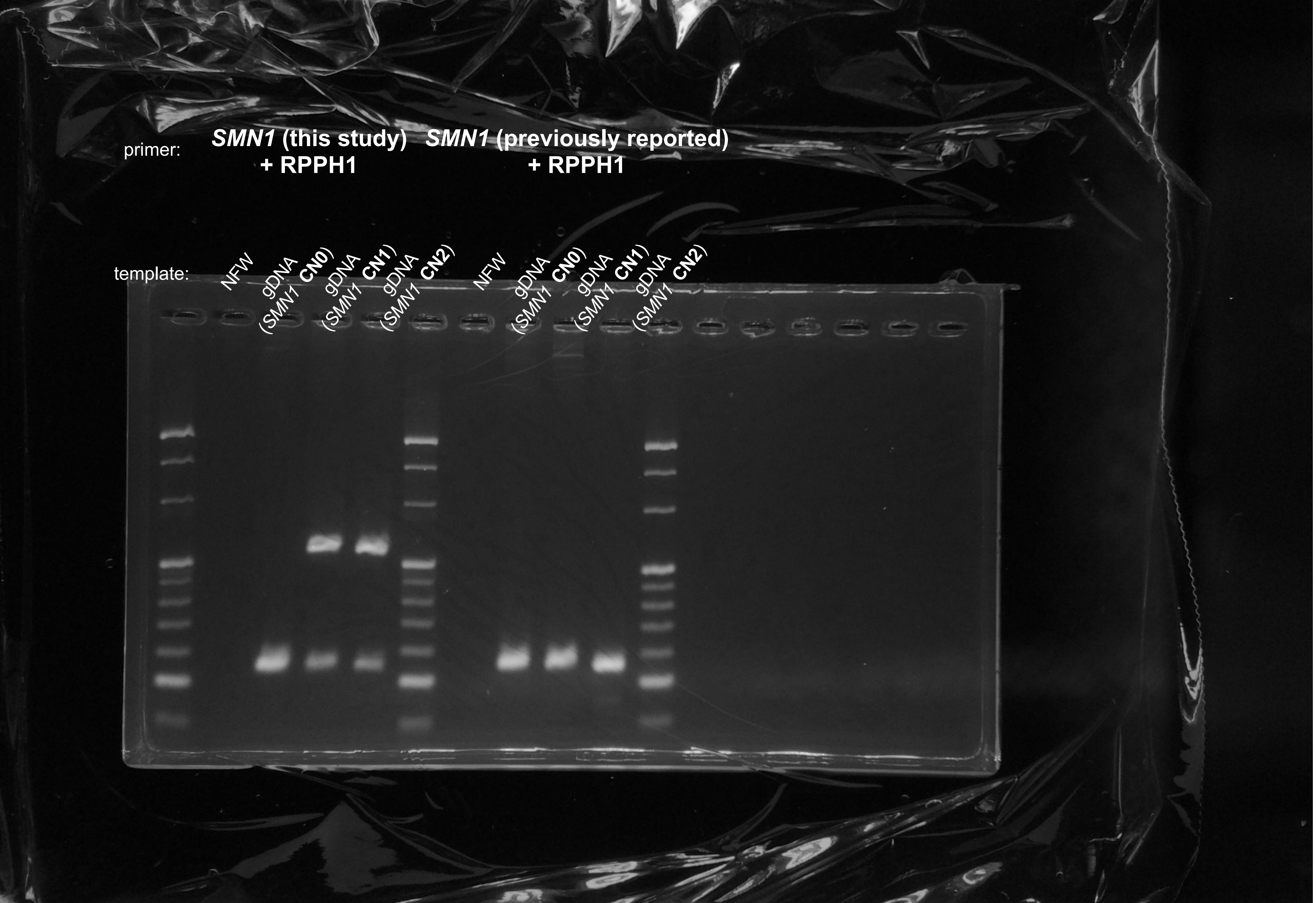

The original gel image of Figure 2(b), captured using a FAS-BG LED BOX (with exposure 10).

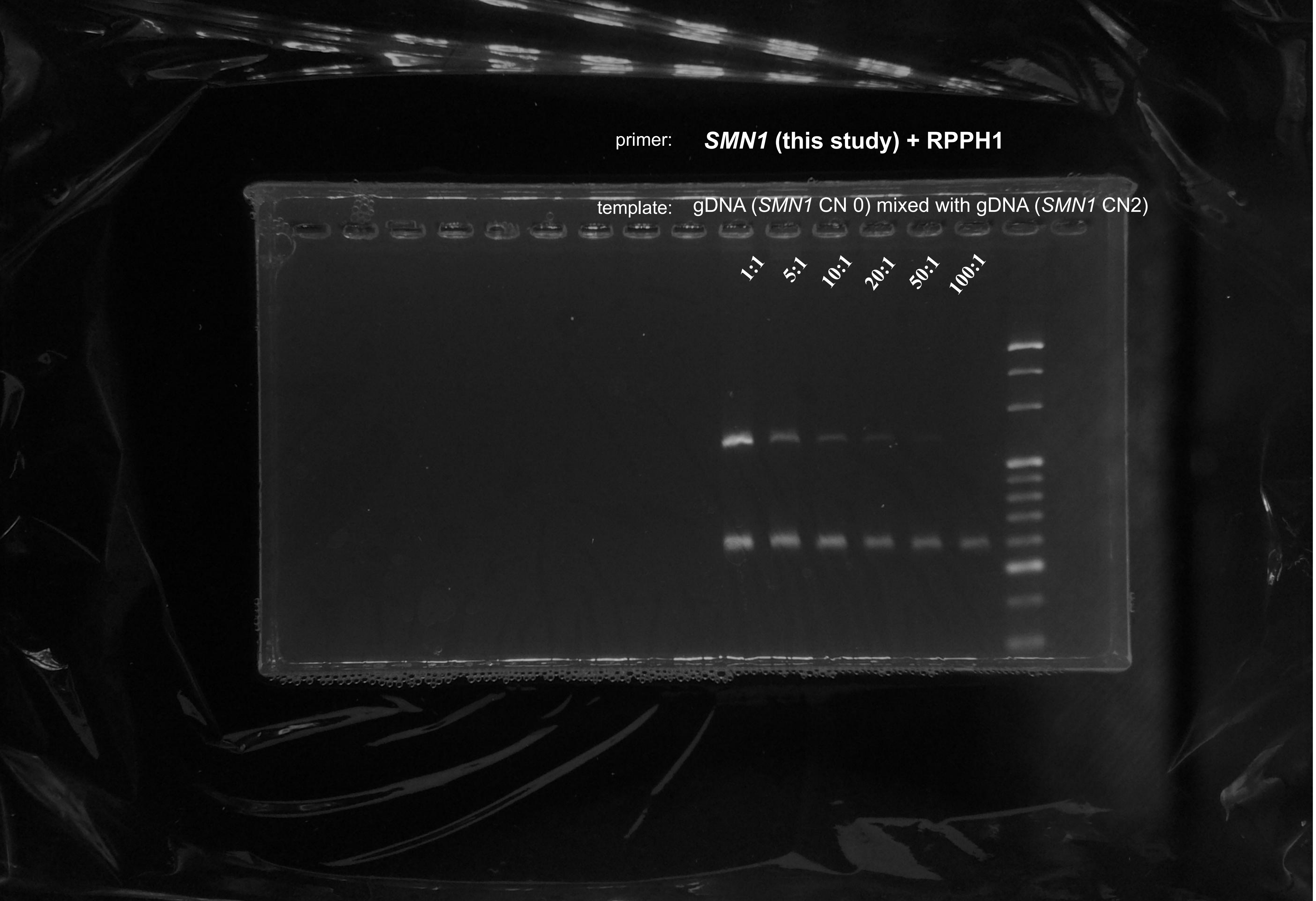

The original gel image of Figure 2(c), captured using a FAS-BG LED BOX (with exposure 10).

The mixing ratios of gDNA (*SMN1* CN 0) and gDNA (*SMN1* CN2) were 1:1, 5:1, 10:1, 20:1, 50:1, and 100:1.

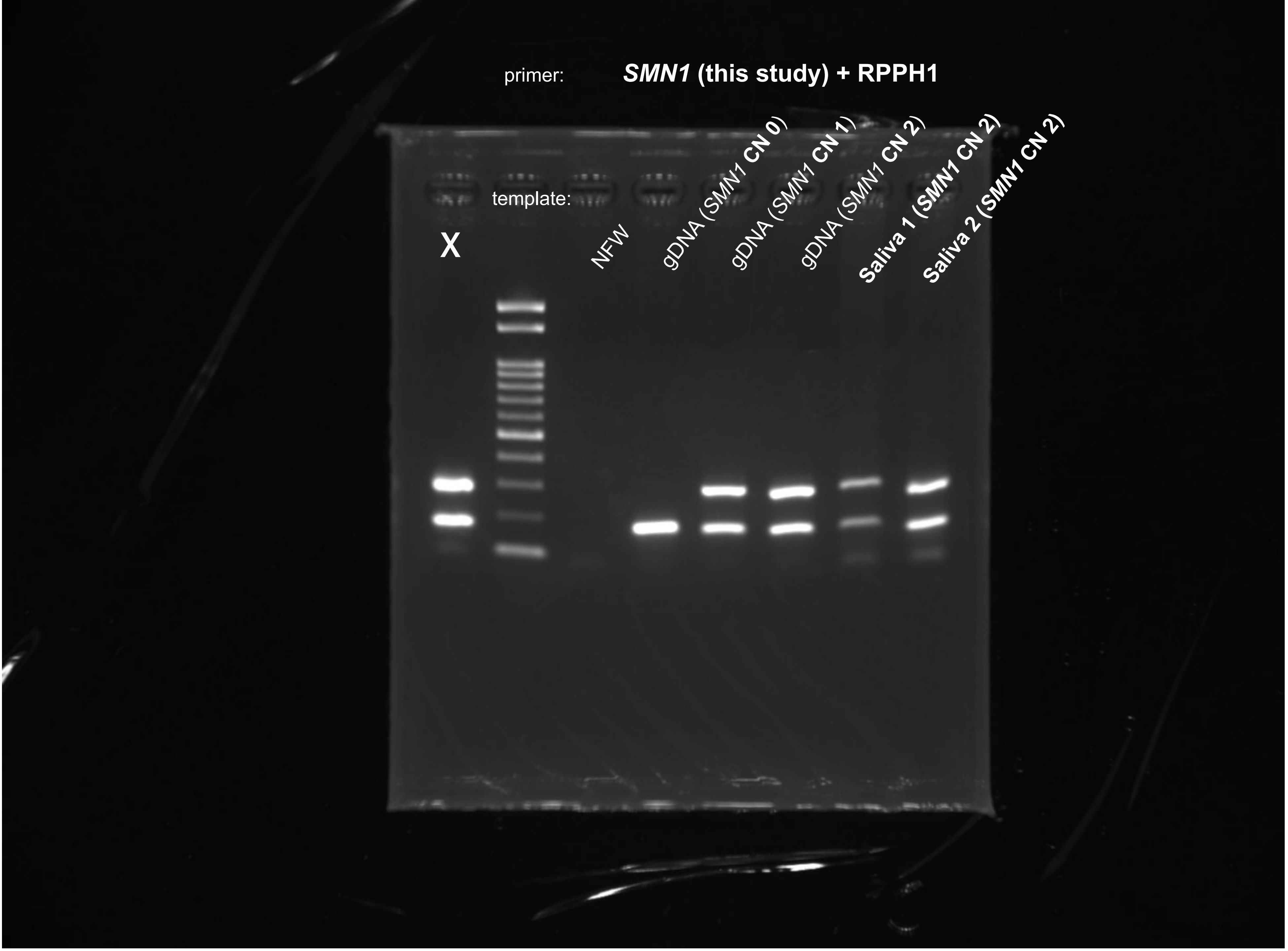

The original gel image of Figure 3(b), captured using a FAS-BG LED BOX (with exposure 10).
