# Supplementary material for: A rapid and easy-to-use spinal muscular atrophy screening tool based on primers with high specificity and amplification efficiency for SMN1 combined with single-stranded tag hybridization assay
Source: PLoS One. 2024 Aug 1;19(8):e0308179. doi: 10.1371/journal.pone.0308179 (PMC11293695; doi:10.1371/journal.pone.0308179)
Supplement: Attachment — Submitted filename: Response to Reviewers_v1.docx [file pone.0308179.s005.docx]

**Responses to Reviewers**

・We appreciate the reviewers’ comments and suggestions, which have been immensely beneficial to our manuscript. We have corrected our paper in line with the reviewers’ suggestions, which can be observed in our responses below.

・Regarding references, ref 3 has been updated to the appropriate bibliography as it contained different information than the intended bibliography. In addition, a paper reporting on the STH assay has been added as new ref 14, so the following reference numbers are off by one.

**To Academic editor:**

1. The control primers utilized (ref 13) should be better defined (is just the same primer sequence or also the fluorophores). In addition, there should be a graphic representation of the genes analyzed and the approximate location of the primers selected and control primers.

Reply:

We apologize for the inadequate description in the manuscript.

In the Methods section, we have added that the primers for *SMN1* with exactly the same sequences as in ref 13 were also used in this study, and that the fluorescent probe (TaqMan MGB probe) with the same sequence as in ref 13 and a different dye (FAM) was used in this study.

We have also added a schematic overview of the genes analyzed and the approximate location of the primers selected and control primers in the new S1 Figure.

2. The authors should better specify what conditions have been used to run the agarose gels. 100V alone do not define the size of the gel, V x cm is usually the unit utilized to express the voltage utilized to run an agarose gel.

Reply:

We apologize for the confusion caused by using the wrong unit. We have corrected it to V/cm as you indicated.

3. Figure 4 is difficult to read and should be enlarged or modified, especially the PCR in (a) and panels c,d,e.

Reply:

We have updated Figure 4 to make it easier to see.

4. STH assays in Figures 3 and 4 should have an enhanced contrast/luminosity to clearly visualize the bands.

Reply:

We appreciate you pointing this out to us.

We have re-shot the samples or adjusted the brightness and contrast appropriately to make the blue lines of the STH assay more visible.

**To Reviewer #1**

1. Throughout the manuscript the primers/probe set designed by the authors were compared to screening primers used in reference 13, from this point forward referenced as German primers/probe set. The authors made no mention of any changes they may have made to the German primers, suggesting that no changes were made or they were just no mentioned in the manuscript. This presents a problem because the German probe contains Rhodamine 6 fluorophore which has extensive crosstalk with the NED fluorophore on their RPPH1 probe which could skew results and make them difficult to quantify.

Reply:

We apologize for the inadequate description in the manuscript.

In the Methods section, we have added that the primers for *SMN1* with exactly the same sequences as in ref 13 were also used in this study, and that the fluorescent probe (TaqMan MGB probe) with the same sequence as in ref 13 and a different dye (FAM) was used in this study.

2. Additionally, in figure 2b, the authors comparing simultaneous amplification of their SMN1 primer set and RPPH1 with the German SMN1 primer set and RPPH1. There is only a 20bp difference between German SMN1 PCR product and RPPH1, is a 2% agarose gel with a 25 minute run time enough to resolve these two bands in the same well.

Reply:

We appreciate you bringing this very important issue to our attention. To resolve this issue with Figure 2, we performed the same experiments using a new 4% agarose gel and 20 bp ladder (7 V/cm, 60min) and confirmed that the results were the same as in the previous experiments.

3. Figure 3a is small, dimly lit and unclear. Figure 4 c-e (upper) are illegible.

Reply:

We have updated Figures 3 and 4 to make them more visible.

4. I think it's also necessary to include any changes you made to the German primer/probe set used in reference 13. This includes changes made to the probe's fluorophore and the specific tags used for the STH assay.

Reply:

In the Methods section, we have added that the primers for *SMN1* with exactly the same sequences as in ref 13 were also used in this study, and that the fluorescent probe to detect *SMN1/2* (TaqMan MGB probe) had the same sequence as in ref 13 and a different dye (FAM).

We have also added a description of the modifications made to the primers for the STH assay in the Methods section.
